# Carnosic Acid Attenuates TNF-α-Induced Insulin Resistance by Regulating Mitochondrial Function in 3T3-L1 Adipocytes

**DOI:** 10.3390/cimb48070736

**Published:** 2026-07-20

**Authors:** Chia-Yuan Lin, Lok-I Chan, Yu-Hsuan Chang, Meng-Chun Lu, Chia-Wen Tsai

**Affiliations:** 1 Department of Food Science, National Taiwan Ocean University, Keelung City 202, Taiwan; a8760752@mail.ntou.edu.tw; 2 Department of Nutrition, China Medical University, Taichung City 406, Taiwan; camillachan418@gmail.com (L.-I.C.); a0960029658@gmail.com (Y.-H.C.); 3 Department of Clinical Nutrition, China Medical University Hospital, Taichung City 404, Taiwan; 010294@tool.caaumed.org.tw; 4 Neuroscience and Brain Disease Center, China Medical University, Taichung City 406, Taiwan

**Keywords:** carnosic acid, N-acetyltransferase 1, mitochondrial dysfunction, insulin resistance, 3T3-L1 adipocytes

## Abstract

The disruption of mitochondrial homeostasis is a trigger for insulin resistance. The loss of N-acetyltransferase 1 (Nat1) function, an insulin-sensitivity gene, contributes to mitochondrial dysfunction and insulin resistance. Carnosic acid (CA), a diterpene derived from rosemary, has demonstrated an anti-insulin-resistance effect. This study hypothesized that CA protects against TNF-α-induced insulin resistance in 3T3-L1 adipocytes by regulating mitochondrial dynamics, biogenesis, and function via Nat1. 3T3-L1 adipocytes were pretreated with CA for 12 h, followed by co-treatment with TNF-α for an additional indicated duration. Results showed that treatment of 3T3-L1 adipocytes with TNF-α decreases mitochondrial membrane potential (MMP) and PGC-1α protein levels and alters mitochondrial fission/fusion dynamics. Pretreatment with CA improved these effects. In parallel, CA prevented the TNF-α-induced reduction in Nat1 protein and improved insulin signaling by suppressing the phosphorylation of insulin receptor substrate-1 (IRS-1) at serine^307^, while restoring the phosphorylation of IRS-1 at tyrosine^628^ and Akt. Moreover, transfection with Nat1 siRNA inhibited the protective effect of CA against TNF-α-induced reductions in MMP, PGC-1α, and insulin signaling. In conclusion, CA ameliorated TNF-α-induced insulin resistance by reducing mitochondrial dysregulation by Nat1.

## 1. Introduction

Mitochondria are responsible for aspects of cellular homeostasis, such as energy metabolism. The generation of mitochondrial membrane potential (MMP) is mediated by proton pumps in complexes I, III, and IV and is an essential component of energy storage during oxidative phosphorylation. Subsequently, the proton gradient generates the membrane transport potential for hydrogen ions, which is harnessed to make ATP [1]. The collapse of MMP could induce mitochondrial impairment and insulin resistance [2]. For instance, the mitochondrial fraction of intermyofibrillar protein from obese individuals exhibits decreased levels of electron transport chain complexes I and III and ATP synthase [3]. Moreover, the mitochondrial oxidative activity and ATP synthesis in skeletal muscle are decreased in patients with severe insulin resistance [2,4]. In the tumor necrosis factor (TNF-α)-induced insulin-resistant model of 3T3-L1 adipocytes, TNF-α treatment led to decreased MMP [5,6]. Thus, maintaining mitochondrial health is essential for developing therapeutic strategies against insulin resistance.

Mitochondrial dynamics and biogenesis are crucial for regulating mitochondrial homeostasis, quality, and quantity [7]. Mitochondrial fission is controlled by fission proteins, including dynamin-related protein 1 (DRP1) and fission 1 (FIS1). The mitofusin 2 (MFN2) and optic atrophy 1 (OPA1) proteins are responsible for the mitochondrial fusion stage [8]. Additionally, the peroxisome proliferator-activated receptor gamma coactivator 1-alpha (PGC-1α), a transcriptional coactivator, is the primary regulator of mitochondrial biogenesis [9]. Perturbations in mitochondrial dynamics and biogenesis are strongly associated with mitochondrial impairment and the onset of insulin resistance. Mitochondrial dynamics and biogenesis are altered in *ob*/*ob* mice, as indicated by decreased MFN2 and OPA1 proteins, increased DRP1 protein, and suppressed PGC-1α mRNA [10]. Treatment of 3T3-L1 adipocytes with TNF-α led to upregulation of FIS1 and DRP1 proteins, downregulation of OPA1 protein, and suppression of the PGC-1α mRNA level [6,11]. However, PPARγ agonist punicic acid, when treated with 3T3-L1 adipocytes, could reverse these effects of TNF-α [6].

N-acetyltransferase 1 (Nat1) and 2 (Nat2) have been identified as insulin-sensitivity genes [12]. The genome-wide association study (GWAS) analysis revealed that the human Nat2 polymorphism was strongly associated with decreased insulin sensitivity [12]. In 3T3-L1 adipocytes, knockout of Nat1 (ortholog of human Nat2) reduced insulin-stimulated glucose uptake, whereas overexpression of Nat1 had the opposite effect. Similarly, Nat1-null mice also showed elevated fasting blood glucose and insulin levels, along with decreased insulin sensitivity [12]. Additionally, Nat1 is considered a key regulator of mitochondrial dynamics and biogenesis. In 3T3-L1 adipocytes, Nat1 silencing significantly enhanced DRP1-dependent mitochondrial fragmentation and reduced the mRNA and protein of PGC-1α [13]. Moreover, knockdown of Nat1 in 3T3-L1 adipocytes led to a significant decrease in MMP [13].

Carnosic acid (CA), a diterpene component, is isolated from rosemary (*Salvia rosmarinus*) leaves and has been shown to display anti-inflammatory, anti-diabetic, and anti-obesity properties [14,15,16]. In 3T3-L1 preadipocytes and in a C57BL/6 J mouse model, treatment with CA mitigates bisphenol A-induced lipid accumulation via regulating adipocyte differentiation, adipogenesis, and lipolysis [16]. Another study found that CA treatment counteracts the palmitate-triggered insulin resistance in 3T3-L1 adipocytes [15]. Recently, our study further indicated that CA prevents 3T3-L1 adipocytes from TNF-α-induced impairment of mitochondrial biogenesis and glucose uptake by promoting PGC-1α and ubiquitination of PARIS by parkin [17]. Although CA has been reported to regulate mitochondrial biogenesis and improve insulin resistance, no studies have elucidated its anti-insulin-resistance role via Nat1-mediated regulation of mitochondrial function and insulin signaling. In the present study, we hypothesize that the protective effects of CA on TNF-α-triggered insulin resistance occur through the modulation of mitochondrial dynamics, biogenesis, and function of Natl in 3T3-L1 adipocytes.

## 2. Materials and Methods

### 2.1. Materials

CA was purchased from Cayman Chemical Company (89820; Ann Arbor, MI, USA). Insulin was purchased from Merck KGaA (407709; Darmstadt, HE, Germany). Dulbecco’s Modified Eagle Medium (DMEM) (12100-046) and penicillin/streptomycin (15140-122) were purchased from Gibco (Thermo Fisher Scientific Inc., Waltham, MA, USA). Fetal calf serum was purchased from Hyclone (SH30087.03; Cytiva, Logan, UT, USA). Dexamethasone (D4902), dimethyl sulfoxide (DMSO) (D8418), 3-isobutyl-1-methyl-xanthine (I5879), protease inhibitor cocktail tablets (SI-S8830), phosphatase inhibitor cocktail 3 (SI-P0044), and polyvinylidene fluoride membrane (NEF1002001PK) were purchased from MilliporeSigma (Merck KGaA, Darmstadt, HE, Germany). In addition, phospho-insulin receptor substrate-1 (IRS-1) tyrosine 628 (p-IRS-1Tyr^628^) and PGC-1α primary antibodies were purchased from Merck KGaA (Darmstadt, HE, Germany). All primary antibodies were purchased from Santa Cruz Biotechnology (Dallas, TX, USA). 3, 3-Dihexyloxacarbocyanine iodide (DiOC_6_) dye was purchased from Calbiochem (53213-82-4; Merck KGaA, Darmstadt, HE, Germany).

### 2.2. Cell Culture, Differentiation, and Treatment

**Figure 1:**
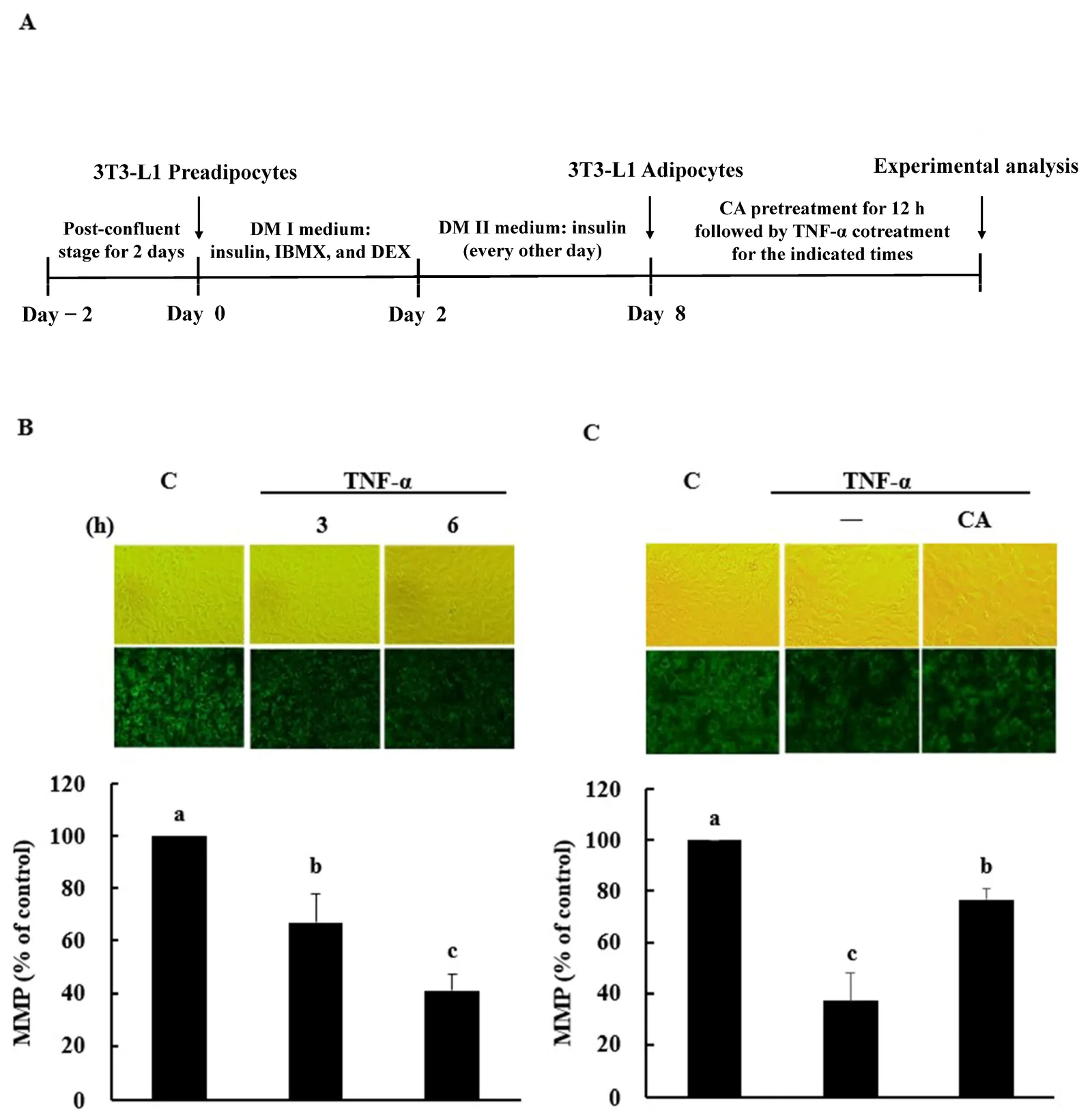
CA improved the TNF-α-induced loss of MMP. (**A**) The process of preadipocyte differentiation and combined treatment with CA and TNF-α. (**B**) 3T3-L1 adipocytes were exposed to TNF-α (5 ng/mL) for 3 and 6 h, respectively. For the control treatment, adipocytes were treated with 0.01% sterile double-distilled water (control, C) for 6 h. (**C**) After 10 μM CA pretreatment for 12 h, 3T3-L1 adipocytes were co-cultured with TNF-α (5 ng/mL) for an additional 6 h. For the control treatment, adipocytes were treated with 0.1% DMSO and 0.01% sterile double-distilled water (control, C) for 18 h. The MMP level was determined using DiOC_6_ staining. The MMP results were calculated by dividing the green fluorescence intensity by the number of cells. The value obtained for the control group was regarded as 100%. One representative image out of three independent experiments is shown. Values are means ± SD (n = 3). Different letters between groups indicate significant differences (*p* < 0.05).

The murine 3T3-L1 preadipocytes cells (BCRC Number: 60159) were purchased from the Bioresource Collection and Research Center (BCRC, Hsin-Chu, Taiwan), and cultured in DMEM supplemented with 10% fetal calf serum, sodium bicarbonate (18 mM), and antibiotics (1 × 10^5^ units/L penicillin and 100 mg/L streptomycin) under a humidified atmosphere of 5% CO_2_ at 37 °C following our previous method by Lin et al. [17]. After 2 days, 3T3-L1 preadipocytes reached confluence and were then cultured in fresh differentiation medium (DM) I, containing insulin (10 μg/mL), 0.5 mM 3-isobutyl-1-methyl-xanthine (IBMX), and 0.5 μM dexamethasone for an additional 48 h. Then, preadipocytes were exposed to fresh DM II with insulin (10 μg/mL), and the DM II was replaced every other day. On day 8, preadipocytes were converted into mature adipocytes (Figure 1A). CA was dissolved in DMSO, TNF-α was dissolved in sterile double-distilled water, and insulin was dissolved in 0.01 N HCl. Our previous study showed that CA pretreatment at 10 μM attenuated TNF-α-induced inflammatory gene expression in 3T3-L1 adipocytes [18]. In this study, a concentration of 10 μM CA was selected for subsequent experiments. 3T3-L1 adipocytes were pretreated with 10 μM CA and then co-treated with 5 ng/mL TNF-α for the indicated times (Figure 1A). The adipocytes in the control group were treated with 0.1% DMSO. In the insulin signaling experiment, adipocytes were incubated with 10 nM insulin for 30 min before harvesting.

### 2.3. Mitochondrial Membrane Potential Assay

DiOC_6_, a green-fluorescent dye, is utilized for the staining of mitochondria in live cells [19]. After treatment, adipocytes were rinsed twice with warm phosphate-buffered saline and then incubated with DiOC_6_ dye for 30 min. The green fluorescence was detected in changes in MMP under fluorescence microscopy. The signal intensity of the fluorescence image was quantified using an Image-Pro Plus 6.0 (Media Cybernetics, Inc., Bethesda, MD, USA). The number of cells was quantified using ImageJ software. The MMP results were calculated by dividing the green fluorescence intensity by the number of cells. The value obtained for the control group was regarded as 100%.

### 2.4. Protein Extraction and Western Blotting

After treatment, adipocytes were rinsed with cold phosphate-buffered saline and were then lysed in an RIPA buffer (BB-40; BIOKIT Biotechnology Inc., Miaoli, Taiwan), containing 1% protease inhibitor and 1% phosphatase inhibitor. After centrifugation at 15,000 rpm for 20 min at 4 °C, the supernatant was obtained as total protein. The concentration of protein from each sample was determined using a Bio-Rad Protein Assay Dye Reagent Concentrate (5000006; Bio-Rad, Hercules, CA, USA).

All proteins were loaded with 10 μg of total cellular protein onto 10% SDS-PAGE gels, except for Nat1, which was loaded with 20 μg. After electrophoresis, SDS-PAGE gels were transferred to polyvinylidene fluoride membranes, and the nonspecific sites on the membranes were blocked in 5% skim milk containing 25 mM Tris and 150 mM NaCl, overnight at 4 °C. Next, each membrane was individually incubated with primary antibodies against Nat1 (sc-137204), OPA1 (sc-393296), MFN2 (sc-515647), DRP1 (sc-271583), FIS1 (sc-376447), PGC-1α (ST1202), IRS-1 (sc-559), p-IRS-1Tyr^628^ (09-433), p-IRS-1Ser^307^ (sc-33956), Akt (sc-55523), phospho-Akt (sc-101629), and glyceraldehyde 3-phosphate dehydrogenase (GAPDH) (sc-47724) at 4 °C overnight. All primary antibodies were diluted at 1:1000, except for the Nat1 primary antibody, which was diluted at 1:500. Each membrane was further incubated with HRP-conjugated secondary antibody at room temperature for 1 h (antibody dilution, 1:1000). Protein bands were visualized using a luminescent image analyzer (LAS-4000, FUJIFILM, Tokyo, Japan).

### 2.5. Transient Transfection of Small Interfering RNA

Primer sequences for siRNA were designed as follows: for murine Nat1 siRNA (siNat1 #1) (5′-GGACGAUGUAGAUCUGGUU-3′) and siNat1 #2 (5′-CAAGAACUCAGUGAAUAAA-3′) (Thermo Fisher Scientific, Waltham, MA, USA). According to published research [17], the reaction mixture solution, containing 50 μL of OptiMEM I-reduced serum media, 29 μL of siRNA primer (final concentration: 100 nM), and 1 μL of Lipofectamine RNAiMAX transfection reagent (56532; Invitrogen, Thermo Fisher Scientific, Waltham, MA, USA), was mixed and reacted for 20 min at room temperature. Meanwhile, 3T3-L1 adipocytes were detached using 0.25% trypsin and centrifuged at 1400 rpm for 5 min. The resulting cell pellets were then resuspended in fresh MEM medium. Moreover, the reaction mixture solution was gently mixed with suspended adipocytes at a density of 0.58 × 10^6^ cells, seeded into 12-well plates, and then incubated in a humidified incubator at 37 °C and 5% CO_2_. After transfection for 24 h, 3T3-L1 adipocytes were pretreated with or without CA at 10 μM and were then co-treated with 5 ng/mL TNF-α for an additional 6 h (for Natl protein, PGC-1α protein, and MMP assay) or 24 h (for insulin signaling-related proteins). Insulin was added to the 3T3-L1 adipocytes for 30 min before collection for insulin signaling in the experiment.

### 2.6. Statistical Analysis

All data were presented as mean ± SD. Values were statistically analyzed using one-way ANOVA with Tukey’s test in the Statistical Analysis System (SAS Institute Inc., Cary, NC, USA). For the results of insulin signaling-related proteins, Student’s *t*-test was performed between the two groups. A *p*-value < 0.05 was considered statistically significant.

## 3. Results

### 3.1. CA Alleviated the Reduction in MMP Caused by TNF-α in 3T3-L1 Adipocytes

The DiOC_6_ staining was used to determine the change in MMP upon the treatment of 3T3-L1 adipocytes with TNF-α in the presence or absence of CA. In Figure 1B, TNF-α treatment decreased the MMP in a time-dependent manner. Compared with that of the control cells, treatment with TNF-α for 3 or 6 h caused a reduction in MMP by 49.2% and 59.1%, respectively. Pretreatment of CA inhibited TNF-α-reduced MMP by nearly 106.7%, compared with TNF-α alone (Figure 1C). These results suggest that CA may protect against TNF-α-induced MMP loss.

### 3.2. CA Affects the Alteration of Mitochondrial Dynamin-Related Proteins in TNF-α-Treated 3T3-L1 Adipocytes

**Figure 2:**
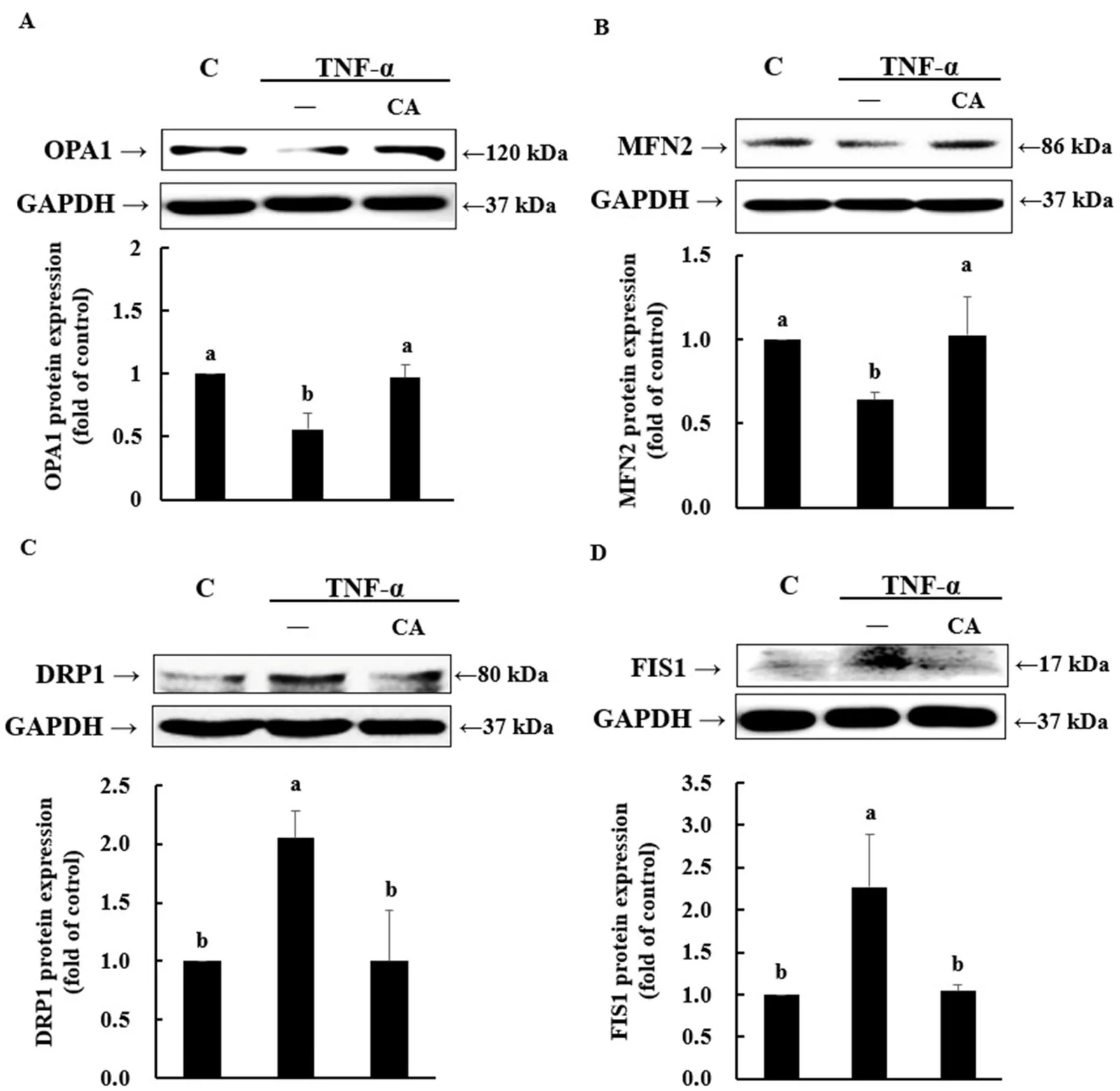
CA-reversed TNF-α caused the induction of mitochondrial fission proteins and a reduction in mitochondrial fusion proteins. 3T3-L1 adipocytes were pretreated with 10 μM CA for 12 h and then co-treated with TNF-α (5 ng/mL) for an additional 2 h. For the control treatment, adipocytes were treated with 0.1% DMSO and 0.01% sterile double-distilled water (control, C) for 14 h. Expressions of mitochondrial dynamin-related proteins were determined by using Western blotting. (**A**) OPA1, (**B**) MFN2, (**C**) DRP1, and (**D**) FIS1. Normalization of Western blots was ensured by GAPDH. The expression of the control group was regarded as 1.0. One representative immunoblot out of three independent experiments is shown. Values are means ± SD (n = 3). Different letters between groups indicate significant differences (*p* < 0.05).

To investigate the preventive effects of CA on TNF-α-induced changes in mitochondrial dynamics, the levels of mitochondrial dynamin-related proteins were assessed by Western blotting. In 3T3-L1 adipocytes treated with TNF-α alone, the protein expressions of OPA1 and MFN2 were decreased, while the protein expressions of DRP1 and FIS1 were increased. However, pretreatment with CA inhibited TNF-α-induced alterations in these proteins (Figure 2). Therefore, CA may protect mitochondria by regulating the mitochondrial fission/fusion dynamics.

### 3.3. CA Reversed the TNF-α-Induced Reduction in Nat1 Protein Expression in 3T3-L1 Adipocytes

**Figure 3:**
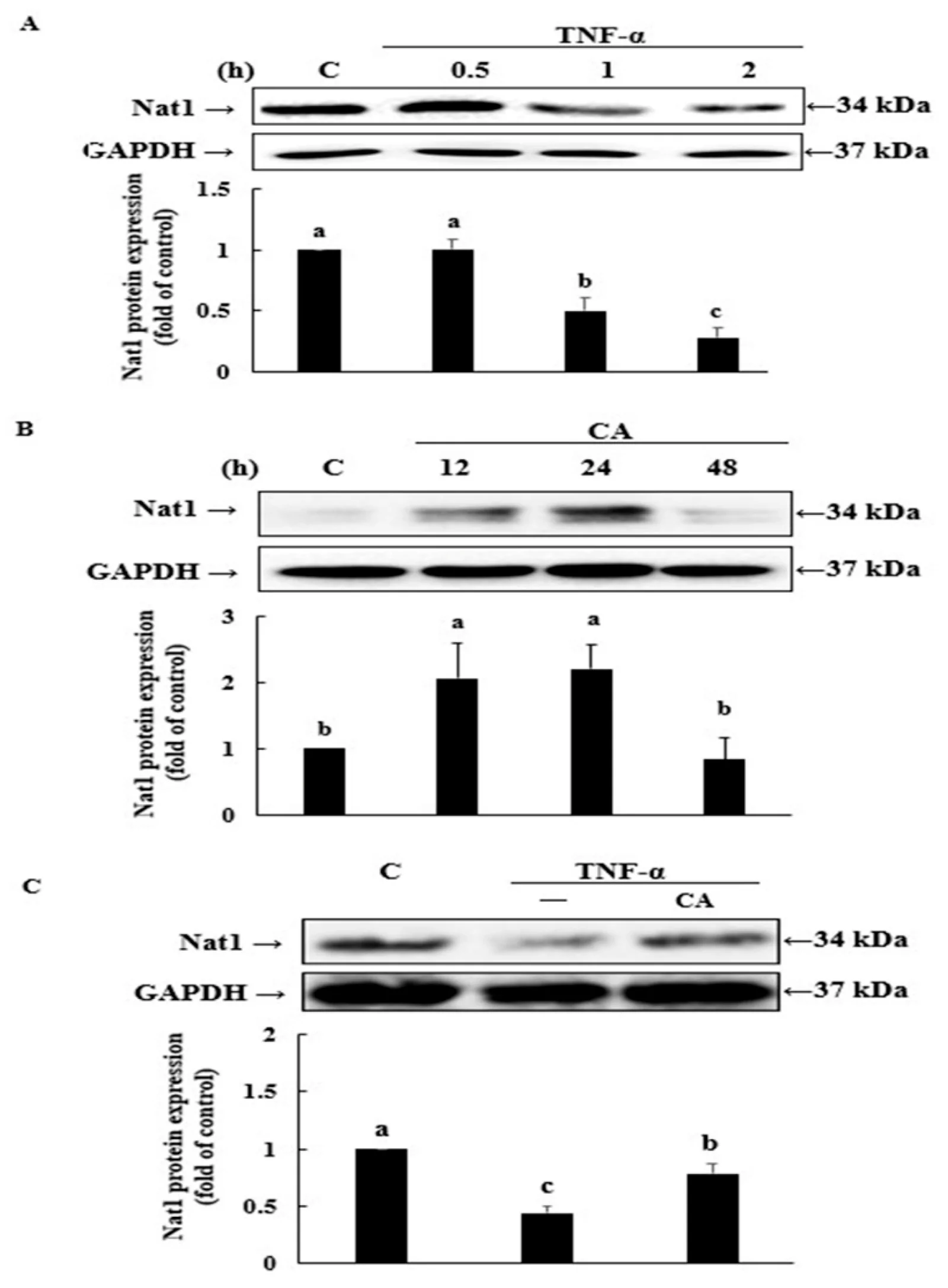
CA-restored TNF-α caused the inhibition of the Nat1 protein. (**A**) 3T3-L1 adipocytes were cultured with TNF-α 5 ng/mL for 0.5, 1, and 2 h. For the control treatment, adipocytes were treated with 0.01% sterile double-distilled water (control, C) for 2 h. (**B**) 3T3-L1 adipocytes were cultured with 10 μM CA for 12, 24, and 48 h. For the control treatment, adipocytes were treated with 0.1% DMSO (control, C) for 48 h. (**C**) 3T3-L1 adipocytes were pretreated with 10 μM CA for 12 h, followed by co-treatment with TNF-α 5 ng/mL for an additional 2 h. For the control treatment, adipocytes were treated with 0.1% DMSO and 0.01% sterile double-distilled water (control, C) for 14 h. Protein expression of Nat1 was determined using Western blotting. Normalization of Western blots was ensured by GAPDH. The level in the control group was regarded as 1.0. One representative immunoblot out of three independent experiments is shown. Values are means ± SD (n = 3). Different letters between groups indicate significant differences (*p* < 0.05).

To confirm the effect of TNF-α on Nat1 protein expression, 3T3-L1 adipocytes were treated with 5 ng/mL TNF-α for 0.5, 1, and 2 h. As presented in Figure 3A, treatment with TNF-α resulted in a marked decrease in Nat1 protein levels at 1 and 2 h. We also found that 2 h TNF-α treatment decreased Nat1 protein levels by 72%. In addition, 3T3-L1 adipocytes were exposed to 10 μM CA for 12, 24, and 48 h to investigate the impact of CA on Nat1 protein expression. The results showed that CA treatment for 12 and 24 h upregulated Nat1 protein expression. However, Nat1 levels returned to baseline following 48 h of CA treatment (Figure 3B). To evaluate the preventive effects of CA against TNF-α-induced downregulation of the Nat1 protein, 3T3-L1 adipocytes were pretreated with 10 μM CA for 12 h, followed by co-treatment with 5 ng/mL TNF-α for an additional 2 h. CA pretreatment effectively prevented the TNF-α-induced decrease in Nat1 protein expression (Figure 3C).

### 3.4. Nat1 Silencing Prevented CA from Reversing TNF-α-Induced MMP Collapse and Mitochondrial Biogenesis Impairment in 3T3-L1 Adipocytes

**Figure 4:**
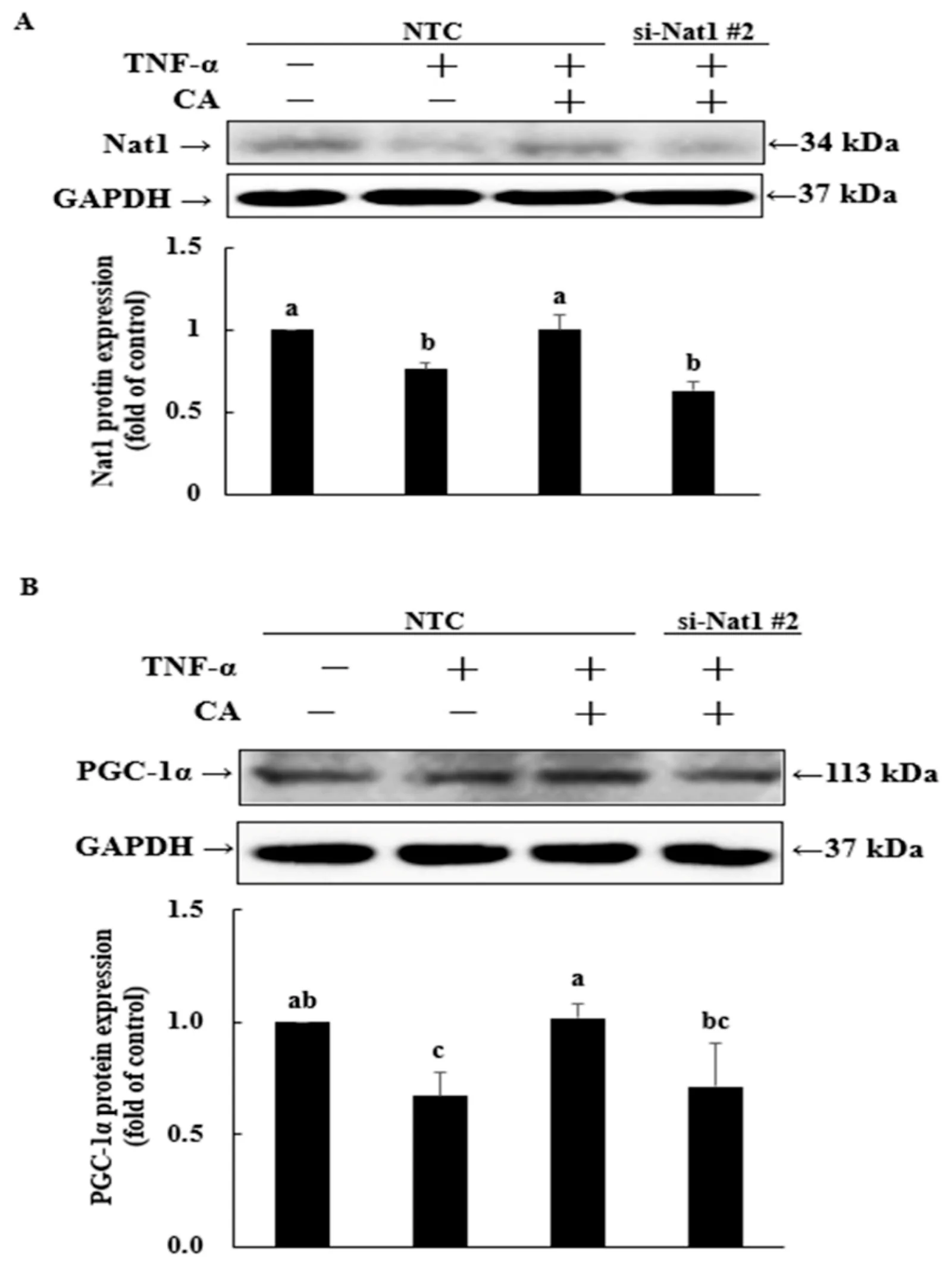
Nat1 siRNA blocked CA’s ability to alleviate TNF-α-induced decreases in PGC-1α protein expression. 3T3-L1 adipocytes were transfected with NTC or Nat1 siRNA #2, respectively. After transfection for 24 h, adipocytes were pretreated with 10 μM CA for 12 h, followed by co-treatment with TNF-α (5 ng/mL) for an additional 6 h. For the control treatment, adipocytes were treated with 0.1% DMSO and 0.01% sterile double-distilled water (control, C) for 18 h. Protein expressions were determined using Western blotting. (**A**) Nat1 and (**B**) PGC-1α. Normalization of Western blots was ensured by GAPDH. The level in the control group was regarded as 1.0. One representative immunoblot out of three independent experiments is shown. Values are means ± SD (n = 3). Different letters between groups indicate significant differences (*p* < 0.05).

To explore whether Nat1 knockdown affects mitochondrial biogenesis and MMP, 3T3-L1 adipocytes were individually transfected with either nontargeting control siRNA (NTC) or Nat1-specific siRNAs. After twenty-four hours, adipocytes were further pretreated with 10 μM CA for 12 h, followed by exposure to 5 ng/mL TNF-α for an additional 6 h. We first observed that transfection of adipocytes with siNat1 #2 significantly reduced Nat1 protein expression, indicating effective knockdown (Supplementary Figure S1). Accordingly, we selected the primer sequence for siRNA-Nat1 #2 for use in subsequent experiments. In NTC-treated 3T3-L1 adipocytes, CA inhibited the TNF-α-induced reduction in Nat1 protein expression. However, in the presence of siRNA-Nat1 #2, this effect of CA was inhibited (Figure 4A).

**Figure 5:**
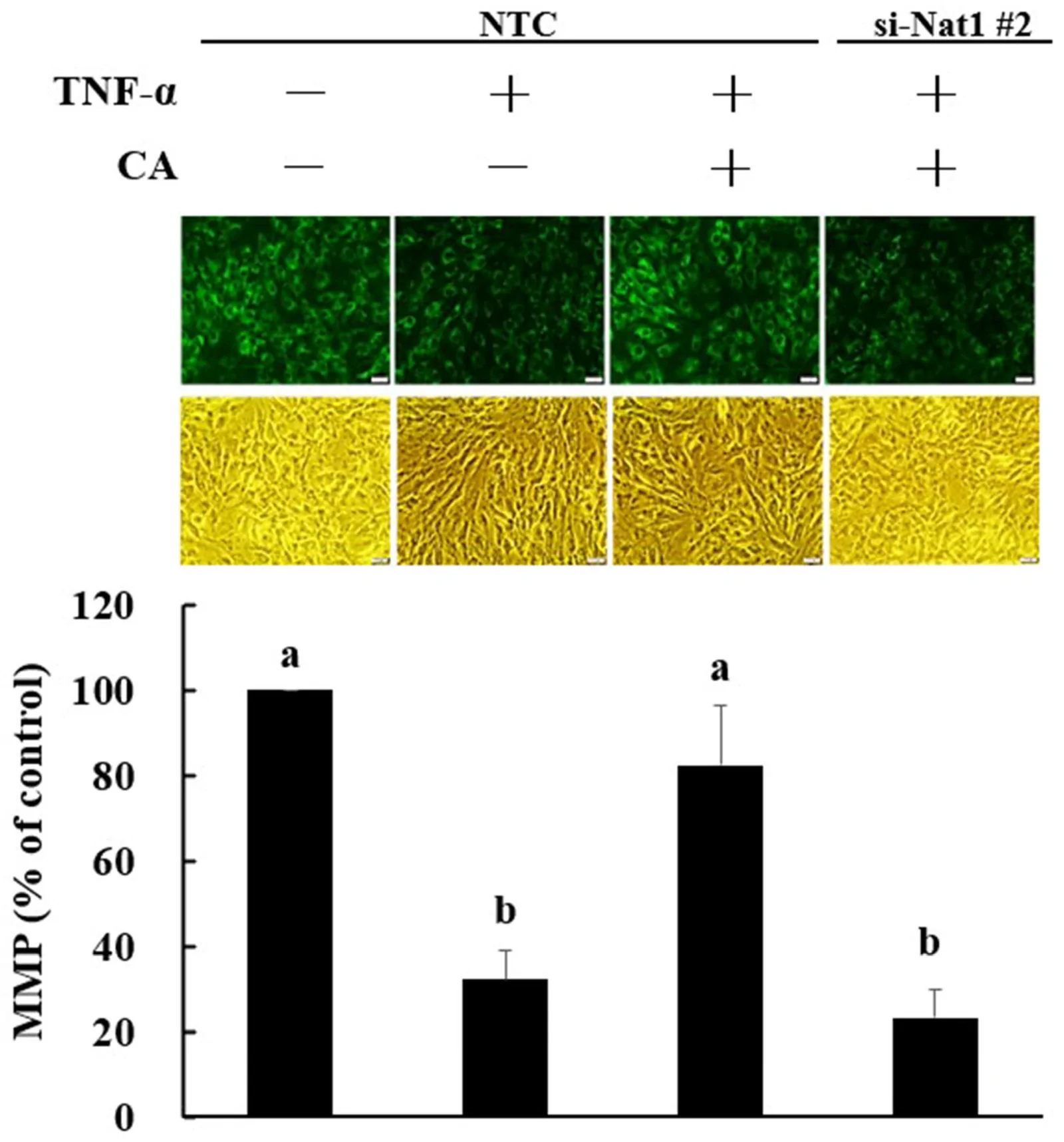
Nat1 siRNA inhibited the ability of CA to improve the TNF-α-induced loss of MMP. 3T3-L1 adipocytes were transfected with NTC or Nat1 siRNA #2, respectively. After twenty-four hours, adipocytes were pretreated with 10 μM CA for 12 h and were then co-treated with TNF-α (5 ng/mL) for an additional 6 h. For the control treatment, adipocytes were treated with 0.1% DMSO and 0.01% sterile double-distilled water (control, C) for 18 h. The level of MMP was determined using DiOC_6_ staining. The results of MMP were calculated by dividing the green fluorescence intensity by the number of cells. The value obtained for the control group was regarded as 100%. The value obtained for the control group was regarded as 100%. One representative image out of three independent experiments is shown. Values are means ± SD (n = 3). Different letters between groups indicate significant differences (*p* < 0.05).

In the experiment of mitochondrial biogenesis, in 3T3-L1 adipocytes treated with NTC, TNF-α treatment inhibited the PGC-1α protein expression, whereas CA pretreatment reversed the effect. Moreover, silencing Nat1 with Nat1 siRNA #2 abolished the protective effect of CA against the TNF-α-induced reduction in PGC-1α expression (Figure 4B). In addition, CA was no longer able to markedly reverse TNF-α-induced loss of MMP in 3T3-L1 adipocytes treated with siRNA-Nat1 #2 (Figure 5). Therefore, the upregulation of Nat1 by CA plays an important role in preserving mitochondrial biogenesis and MMP in 3T3-L1 adipocytes.

### 3.5. Silencing of Nat1 Inhibited the Ability of CA to Reverse TNF-α-Induced Impairment of Insulin Signaling in 3T3-L1 Adipocytes

**Figure 6:**
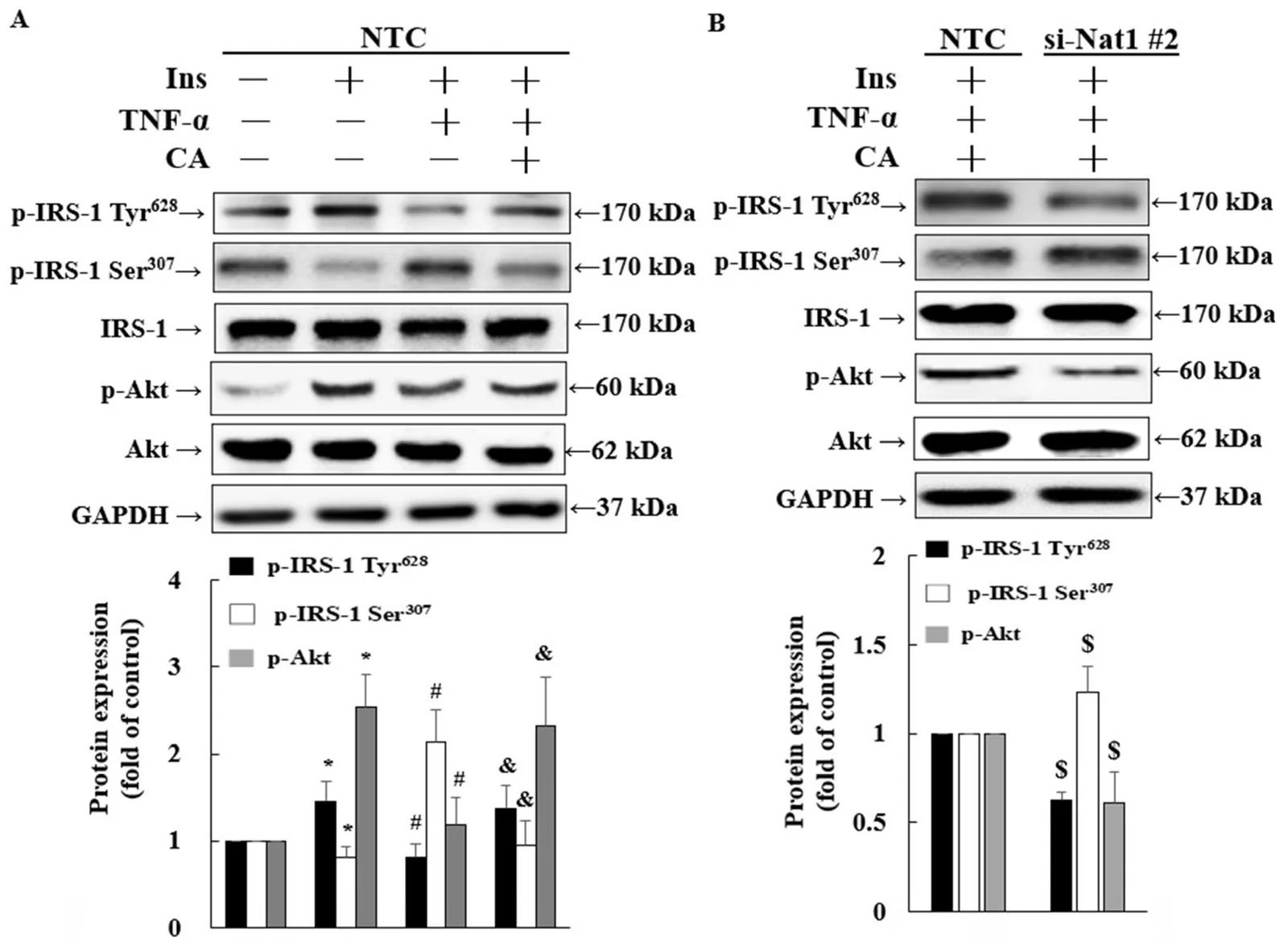
Nat1 siRNA blocked the ability of CA to ameliorate TNF-α-induced impairment of insulin signaling. 3T3-L1 adipocytes were transfected with (**A**) NTC or (**B**) Nat1 siRNA #2 for 24 h. Then, adipocytes were pretreated with 10 μM CA for 12 h, followed by co-treatment with TNF-α (5 ng/mL) for an additional 18 h. Before adipocytes were harvested, insulin (10 nM) was added to the adipocytes for 30 min. For the control treatment, adipocytes were treated with 0.1% DMSO, 0.01% sterile double-distilled water, and 0.01 N HCl (control, C) for 30 h. Protein expressions were determined using Western blotting. Normalization of Western blots was ensured by GAPDH. The level in the control group was regarded as 1.0. One representative immunoblot out of three independent experiments is shown. Values are means ± SD (n = 3). * *p* < 0.05, compared with the NTC + control group. ^#^ *p* < 0.05, compared with NTC + insulin group. ^&^ *p* < 0.05, compared with NTC + insulin + TNF-α group. ^$^ *p* < 0.05, compared with the NTC + insulin + TNF-α + CA group.

Our previous study demonstrated that CA protects 3T3-L1 adipocytes against TNF-α-induced insulin resistance [18]. The present study further investigates whether CA’s preventive effect against insulin resistance is mediated by upregulation of Nat1 protein levels. In NTC-treated 3T3-L1 adipocytes, insulin treatment decreased the phosphorylation of IRS-1 at Ser^307^ and increased the phosphorylation of IRS-1 at Tyr^628^ and Akt. However, these alterations in protein phosphorylation were reversed in 3T3-L1 adipocytes treated with both TNF-α and insulin. Moreover, pretreatment with CA counteracted the effects of TNF-α, suggesting that CA mitigates TNF-α-induced impairment of insulin signaling (Figure 6A). In siRNA-Nat1 #2-treated 3T3-L1 adipocytes, CA failed to significantly reverse TNF-α-induced alterations in the phosphorylation of these proteins (Figure 6B). Thus, the upregulation of Nat1 is essential to the anti-insulin-resistance effect of CA.

## 4. Discussion

*Nat1* deficiency has been known to cause mitochondrial dysfunction and insulin resistance in mice [13,20]. However, it remains unclear whether the protective effects of CA against TNF-α-induced mitochondrial dysfunction and insulin resistance in 3T3-L1 adipocytes are mediated by enhancing the Nat1 protein. The primary novelty of this research is that CA counteracts TNF-α-induced inhibition of mitochondrial fusion proteins (OPA1 and MFN2), mitochondrial biogenesis-related protein (PGC-1α), and membrane potential, as well as the induction of mitochondrial fission proteins (DRP and FIS1). Moreover, CA-induced Nat1 protein expression improves mitochondrial biogenesis and function, as well as insulin signaling, in TNF-α-treated 3T3-L1 adipocytes. Therefore, Nat1 is essential for the anti-insulin-resistance effect of CA by maintaining mitochondrial fission/fusion dynamics, biogenesis, and function.

Mitochondria, important metabolic organelles, participate in insulin signaling in several metabolic tissues, such as adipose tissue [21]. In adipose tissue, mitochondrial dysfunction is the early stage in the pathological mechanism of obesity-associated insulin resistance [22]. Alterations in mitochondrial mass and structure were often observed in adipocytes of *ob*/*ob* mice [23]. Besides alterations in morphology, mitochondrial dysfunction is characterized by increased mitochondrial fragmentation and decreased MMP, mitochondrial respiration, and ATP content. Moreover, TNF-α-treated insulin-resistant 3T3-L1 adipocytes exhibited markedly elevated reactive oxygen species (ROS) production accompanied by inhibition of MMP and cellular ATP formation [11]. Studies have shown that excess ROS leads to mitochondrial dysfunction and insulin resistance [24]. In 3T3-L1 adipocytes, the accumulation of cellular ROS and the induction of MMP collapse were detected upon treatment with TNF-α. By contrast, (−)-Epigallocatechin-3-gallate (EGCG) from green tea counteracted these effects of TNF-α [5]. The result is similar to the findings reported in this study, TNF-α treatment time-dependently decreased MMP. However, this phenomenon was reversed by the pretreatment of CA (Figure 1B,C). It is possible that CA could counteract the TNF-α-triggered loss of mitochondrial function in 3T3-L1 adipocytes, which is related to the scavenging of ROS formation.

Mitochondria are highly dynamic organelles that undergo fission and fusion in healthy cells. This dynamic process continuously maintains mitochondrial shape, distribution, and size [25]. Elevating the fusion process induced by DRP1 and FIS1 promotes mitochondrial fragmentation to generate small individual mitochondria. Promoting the fusion process, driven by the proteins OPA1 and MFN2, results in mitochondrial elongation and the formation of large, interconnected mitochondria [25]. In addition to mitochondrial quantity, mitochondrial fission and fusion dynamics also regulate insulin signaling [26,27]. Defects in mitochondrial proteins involved in fission and fusion result in an imbalance in mitochondrial dynamics, leading to mitochondrial defects and insulin resistance. OPA1 overexpression in alveolar epithelial (A549) cells significantly ameliorated the PM_2.5_-induced reduction in MMP, whereas DRP1 knockdown showed a trend towards increasing MMP [28]. In differentiated murine adipocytes, nitrite treatment increased mitochondrial respiration and glucose uptake by augmenting mitochondrial fusion via increased MFN1 protein and inhibiting DRP1 protein [26]. In the study by Lin et al., overexpression of DRP1 or FIS1 increased mitochondrial ROS, decreased the tubular network of mitochondria, and inhibited insulin-stimulated activation of IRS-1 and Akt phosphorylation in diabetes-susceptible cybrid cells. However, knockdown of DRP1 or FIS1 and treatment with mdivi-1, a DRP1 inhibitor, had an opposing effect [27]. In the present study, TNF-α treatment in 3T3-L1 adipocytes decreased OPA1 and MFN2 fusion protein and increased DRP1 and FIS1 proteins, while CA pretreatment had an opposing effect (Figure 2). Anusree’s study also showed that treatment of 3T3-L1 adipocytes with TNF-α reduced OPA1 and increased FIS1, leading to mitochondrial dysfunction and insulin resistance [6]. Moreover, punicic acid treatment ameliorates TNF-α-induced alterations in mitochondrial dynamics-associated proteins, rescues mitochondrial efficiency, and improves insulin resistance [6]. Therefore, CA-mediated mitochondrial dynamics might play an essential role in regulating mitochondrial quality in adipocytes.

The Nat gene is a phase II enzyme that catalyzes the acetylation of aromatic and hydrazine drugs [29]. Recently, this gene has been considered an insulin sensitivity gene. Using the GWAS approach, a nonsynonymous single-nucleotide polymorphism in human Nat2 [rs1208 (803A>G, K268R)] is highly associated with insulin resistance-related features, such as elevated hemoglobin A1C and fasting glucose [12]. Moreover, Nat1 knockout mice also developed whole-body insulin resistance, which could be attributed to declines in adipose tissue, muscle, and liver [20]. Nat1 deficiency in 3T3-L1 adipocytes was associated with decreased glucose uptake, while Nat1 overexpression had the opposite result [12]. So far, no studies have demonstrated the role of CA on the Nat1 protein in TNF-α-treated 3T3-L1 adipocytes. Our results showed that pretreatment of CA in 3T3-L1 adipocytes reverses TNF-α-induced protein reduction in Nat1 and the impairment of insulin signaling (Figure 3 and Figure 6A). Also, in the presence of Nat1 siRNA, CA could no longer markedly improve the inhibition of Nat1 protein and insulin signaling by TNF-α, indicating that the elevation of Nat1 by CA is required for the prevention of TNF-α-induced impairment of insulin signaling (Figure 4A and Figure 6B). In addition, the study suggested that silencing of Nat1 led to mitochondrial dysfunction, including increased mitochondrial ROS, decreased MMP, and reduced ATP production [13]. Nat1-deficient 3T3-L1 adipocytes showed an increase in mitochondrial fragmentation and DRP1 expression, suggesting that suppression of Nat1 promotes mitochondrial fragmentation via increasing DRP1 [13]. Furthermore, a significant decrease in the mRNA and protein of PGC-1α was observed in Nat1-deficient adipocytes [13]. In this study, we found that CA pretreatment could improve the TNF-α-induced reduction in PGC-1α protein (Figure 4B). Silencing of Nat1 in 3T3-L1 adipocytes blocked CA from restoring the TNF-α-caused reduction in PGC-1α and MMP (Figure 4B and Figure 5). CA may promote mitochondrial biogenesis to protect mitochondrial function in 3T3-L1 adipocytes against TNF-α-induced mitochondrial damage. In addition to its role of PGC-1α in mitochondrial biogenesis, PGC-1α has been shown to positively modulate insulin signaling. Our recent study showed that silencing PGC-1α with siRNA suppressed CA’s ability to restore TNF-α-inhibited insulin signaling and glucose transport translocation [17]. Therefore, the prevention of TNF-α-caused mitochondrial dysfunction and insulin resistance by CA is related to the induction of the Nat1 protein.

In the present study, the induction of Nat1 protein by CA contributed to the amelioration of mitochondrial damage and the restoration of the impaired insulin signaling pathway following TNF-α treatment. However, our study has certain limitations. We evaluated the MMP assay and the protein expression levels related to mitochondrial dynamics and PGC-1α. In the insulin signaling experiments, we examined only the expression of proteins associated with the insulin signaling pathway. To further elucidate the importance of Nat1, future investigations could examine mitochondrial ROS production, ATP levels, and glucose uptake in the presence or absence of Nat1 knockdown.

## 5. Conclusions

CA alleviated TNF-α-induced dysregulation of mitochondrial function by regulating mitochondrial fission/fusion dynamics and biogenesis in 3T3-L1 adipocytes. Importantly, CA was first exhibited to enhance the Nat1 protein. The induction of Nat1 by CA could reverse the reductions in MMP and PGC-1α protein levels and the TNF-α-induced downregulation of insulin signaling in 3T3-L1 adipocytes. These findings shed light on the mechanistic link at the cellular level between Nat1, mitochondrial function, and insulin signaling by CA in TNF-α-treated 3T3-L1 adipocytes. Therefore, CA may offer protective benefits against mitochondrial dysfunction and insulin resistance by inducing Nat1.

## Data Availability

The original contributions presented in this study are included in the article and Supplementary Material. Further inquiries can be directed to the corresponding author(s).

## Supplementary Materials

The following supporting information can be downloaded at https://www.mdpi.com/article/10.3390/cimb48070736/s1.

## Abbreviations

CA, carnosic acid; DiOC_6_, 3, 3-dihexyloxacarbocyanine iodide; DEX, dexamethasone; DMEM, Dulbecco’s modified Eagle medium; DRP1, dynamin-related protein 1; EGCG, (−)-Epigallocatechin-3-gallate; FIS1, fission 1; GWAS, genome wide association study; IBMX, 3-isobutyl-1-methyl-xanthine; IRS-1, insulin receptor substrate 1; MFN2, mitofusin 2; Nat1, N-acetyltransferase 1; Nat2, N-acetyltransferase 2; MMP, mitochondrial membrane potential; OPA1, optic atrophy 1; PGC-1α, peroxisome proliferator-activated receptor gamma coactivator 1-alpha; ROS, reactive oxygen species; Ser^307^, serine 307; Tyr^628^, tyrosine 628; TNF-α, tumor necrosis factor-α.
