# Supplementary material for: Carnosic Acid Attenuates TNF-α-Induced Insulin Resistance by Regulating Mitochondrial Function in 3T3-L1 Adipocytes
Source: Curr Issues Mol Biol. 2026 Jul 20;48(7):736. doi: 10.3390/cimb48070736 (PMC13409672; doi:10.3390/cimb48070736)
Supplement: Supplementary file 1 [file cimb-48-00736-s001.zip › cimb-4389278-supplementary.pdf]

Supplementary Figure S1

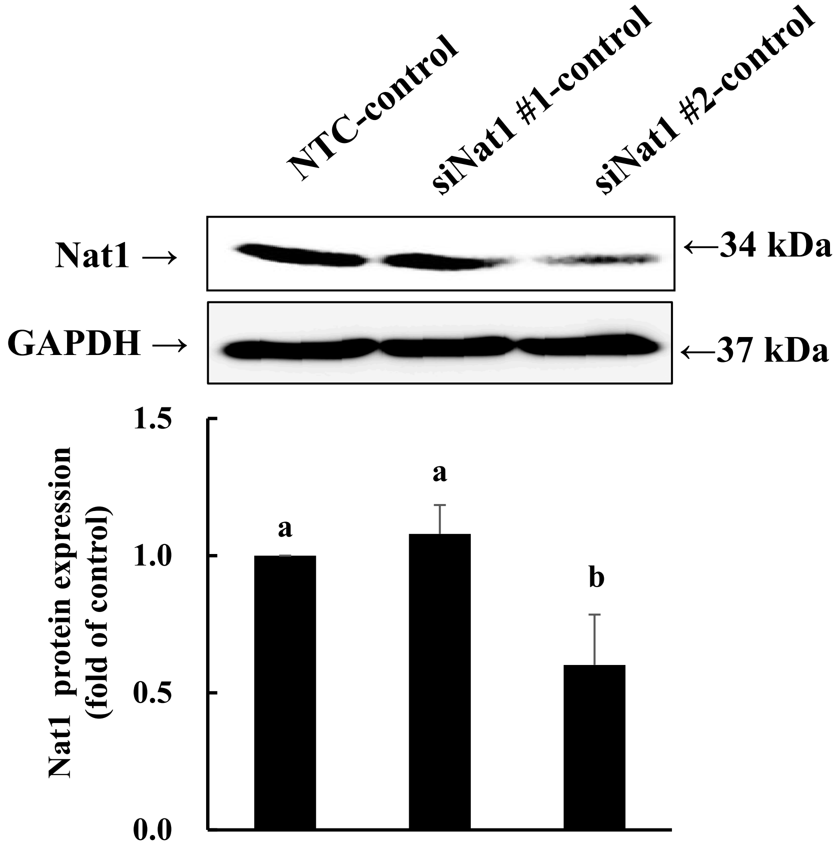

Figure S1. siNat1 #2 led to a significant decrease in Nat1 protein expression. 3T3-L1 adipocytes were individually transfected with either siNat1 #1 or siNat1 #2 for 24 hours. The protein expression of Nat1 was determined by Western blotting. Normalization of Western blots was ensured by GAPDH. The level in control group was regarded as 1.0. One representative immunoblot out of three independent experiments is shown. Values are means ± SD (n = 3). Different letters between groups indicate significant differences ($p < 0.05$).
